# Motivators and Barriers to Accessing Sexual Health Care Services for Transgender/Genderqueer Individuals Assigned Female Sex at Birth

**DOI:** 10.1089/trgh.2018.0022

**Published:** 2019-02-28

**Authors:** Christine Y.W. Harb, Lauren E. Pass, Isabella C. De Soriano, Adelaide Zwick, Paul A. Gilbert

**Affiliations:** ^1^ Kirksville College of Osteopathic Medicine, A.T. Still University, Kirksville, Missouri.; ^2^ Department of Community and Behavioral Health, University of Iowa College of Public Health, Iowa City, Iowa.; ^3^ University of Iowa College of Liberal Arts and Sciences, Iowa City, Iowa.

**Keywords:** disparity, health care, Papanicolaou test, transgender

## Abstract

**Purpose:** Individuals who were assigned female sex at birth (AFAB) but do not conform to the gender binary (i.e., transgender/genderqueer) often do not seek out necessary sexual health care, including Papanicolaou (Pap) tests, placing them at heightened risk of cervical cancer. Despite growing awareness, scant research has focused on the sexual health care experiences of this population in Midwestern and rural states.

**Methods:** We used two approaches to develop a more detailed understanding of factors that determine sexual health care use. Seventeen transgender/genderqueer AFAB individuals completed a quantitative knowledge survey then participated in a semistructured qualitative interview to further elaborate their experiences with sexual health care services as well as the motivators and barriers related to accessing the health care system. We produced descriptive summaries of quantitative data and conducted a thematic analysis of interview transcripts.

**Results:** The sample was mostly young adults, of whom nearly all were white and 65% self-identified as transgender men. Participants displayed good knowledge about human papillomavirus (HPV) and Pap tests; however, 41% rated themselves as “unaware” and 59% rated themselves as “aware but not well informed” about HPV. Fifty-nine percent had ever obtained a Pap test. We identified one facilitating factor (health care provider's role and relationship) and three inhibiting factors (availability of competent care; distress about seeking sexual health care; health care setting characteristics) related to obtaining Pap tests.

**Conclusion:** As this study was conducted in the U.S. Midwest, findings extend the geographic scope of existing knowledge and may inform future risk reduction interventions and clinical practice.

## Introduction

The Papanicolaou (Pap) test is an important cancer screening procedure for people who have cervices. It allows health care providers to detect precancerous and cancerous lesions caused by the human papillomavirus (HPV), which is a sexually transmitted disease. However, not all people who have a cervix are screened according to recommendations, such as the American College of Obstetricians and Gynecologists (ACOG) guidelines,^1^ particularly because not all identify as women.

For instance, transgender men and trans masculine, nonbinary, or gender nonconforming people are individuals who were assigned female sex at birth but identify their gender as male, masculine, or nonbinary on the gender spectrum (hereafter referred to as transgender/genderqueer assigned female sex at birth [AFAB] individuals).^*^ This is different from cisgender individuals, whose gender identity aligns with the sex assigned at birth. Most transgender/genderqueer AFAB individuals do not undergo surgical removal of their natal reproductive organs,^2–4^ and as a result, cancers of these organs, including the cervix, can still develop.^5–7^

The ACOG recommends that transgender/genderqueer AFAB individuals with a cervix follow the same screening guidelines as cisgender women.^1^ Yet studies have found that transgender/genderqueer AFAB people are significantly less likely to be up to date with their Pap tests compared with cisgender women.^5,6,8,9^ This is concerning because transgender/genderqueer AFAB individuals face a disproportionately higher risk of contracting sexually transmitted infections (STI),^8,10–12^ with HPV ranking as the most prevalent STI among transgender/genderqueer AFAB patients, compared with HIV, Chlamydia, bacterial vaginosis, herpes, gonorrhea, and trichomoniasis.^10,11^ Screening for HPV is particularly important because HPV is present in almost 99% of cervical cancers, with two strains (HPV-16 and HPV-18) accounting for the majority (70%) of cervical cancers.^13^

There are many factors that impact Pap test utilization. Notably, physicians' perceptions of risk and knowledge of screening guidelines appear to be instrumental for this population; however, physicians may alter their screening recommendations based on the reported sexual behavior of their transgender/genderqueer AFAB patients. In a study that also included cisgender women, transgender/genderqueer AFAB people who had sex with both males and females were more likely to be up to date with their Pap tests compared with those who exclusively had sex with men.^5^ This finding suggests that physicians are not consistent about following guidelines for recommending Pap tests and may presume that bisexual individuals are at higher risk compared with other patients. Concurrently, it also shows that if physicians offer recommendations for Pap tests or any other screenings, patients are more likely to undergo them.

While transgender/genderqueer AFAB individuals generally believe they should receive regular Pap tests, especially if they have gynecological concerns, many physicians believe that the risk for cervical cancer is low for their transgender/genderqueer AFAB patients who do not have sex with cisgender men and that they do not need to be screened regularly or at all,^14^ which contradicts ACOG recommendations.^1^

The 2015 U.S. Transgender Survey found that 27% of transgender/genderqueer AFAB participants who had a cervix reported a Pap test in the past year, compared with recent surveillance estimates of 43% of cisgender females.^15^ In fact, many providers may be unaware of current recommendations for cervical cancer screenings, particularly as medical school and residency curricula lack education about transgender health care.

A recent cross-sectional survey found that 80% of obstetrics and gynecology providers had not received training on the care of transgender patients.^16^ Only 29% of respondents reported being comfortable with treating female-to-male transgender patients, although almost 90% reported a willingness to conduct a Pap test on this patient population. The willingness to conduct Pap tests, despite low comfort and education level, may contribute to perceptions of incompetence, which in turn may exacerbate feelings of discomfort felt by transgender/genderqueer AFAB patients during the procedure.

Second, regular Pap tests may be more difficult to conduct with this population. Long-term intramuscular androgen administration (hormone therapy) can cause vaginal atrophy and make the Pap test painful, which may add emotional distress to the physical discomfort for some patients.^17^ It may also make successful completion of the test more difficult. Compared with cisgender women, transgender/genderqueer AFAB patients are over 10 times more likely to have an inadequate Pap test, defined as tests which cannot be analyzed in a laboratory due to an insufficient number of cells or a high amount of blood.

Several other factors warrant consideration, including a history of sexual trauma, which may exacerbate anxiety about genital examinations (n.b., this holds for both cis- and transgender persons alike). This could decrease the likelihood of completing Pap tests or STI screenings,^18^ which has been shown in a mixed-methods study of trans men's sexual health needs. Twenty-five percent of those who reported a lifetime STI history did not pursue HIV testing in over 2 years, and 31% had not received gynecological care (including STI screenings) in the past year, even though over a third (38%) of participants had been diagnosed with an STI in the past.^10^

While participants generally rated themselves as highly vulnerable to STIs, there was a gap between perceived risk, past experience, and health care utilization. This suggests that other factors deter transgender/genderqueer AFAB individuals from seeking sexual and reproductive care, such as facing high levels of discrimination from health care providers as well as inconsistent recommendations.^19^

Therefore, in response to both indications of unmet sexual health needs and gaps in knowledge regarding transgender/genderqueer AFAB individuals,^15,20^ this study examined the motivators and barriers to sexual health care services in general, and Pap tests in particular, in a Midwest university town and surrounding areas. The setting is salient, as the state, in which the study took place, is largely rural and characterized by many micropolitan areas with few transgender-specific health care services.

Indeed, the primary motivation for our study was to address a gap in local data (i.e., little research on transgender/genderqueer AFAB populations in Midwest states) as a preliminary step toward local health promotion interventions. We were reluctant to presume that findings from other populations and settings, especially coastal urban centers, would generalize to Midwestern and rural contexts. Given the complexity of the topic, this study used a quantitative survey to assess awareness and knowledge of HPV infection and the Pap test, and qualitative, semistructured interviews to develop a more detailed understanding of salient factors that determine sexual health care use.

## Methods

### Sample

Participants had to meet four eligibility criteria: (1) report having been assigned female sex at birth; (2) report having a cervix and vagina (i.e., no history of hysterectomy or other gender-affirming surgery); (3) currently identify as a transgender male, trans masculine, nonbinary, or gender nonconforming; (4) be 18 years of age or older. An eligibility screening question asked potential participants if they were a transgender man, trans masculine, gender nonbinary, or gender nonconforming (yes/no); however, during the qualitative interview participants were asked to self-identify their gender in an open-ended question. For reporting purposes we summarized responses with the same terms.

Although a major focus of this article is use of Pap tests, the study's overarching aim was to explore use of sexual health care services among transgender/genderqueer AFAB adults (i.e., not minor children and adolescents; not cisgender women) by assessing knowledge of sexual health topics, including HPV, in quantitative surveys and attitudes toward and willingness to access sexual health care services, including Pap tests, in qualitative interviews.

To facilitate recruitment, we used existing university resources; however, our goal was not to obtain a representative sample. Recruitment e-mails were sent to the entire campus through the university's mass e-mail service to inform potential participants of this research project. A recruitment advertisement was also posted in a newsletter at the university's medical center.

Additionally, we used snowball referrals^21^; a study flyer was offered to participants upon completion of the survey and interview, and the study purpose and eligibility criteria were explained. Participants were asked to pass the flyer on to social contacts who might be interested and eligible to participate. Those who were interested in participating contacted the first author through e-mail. Once eligibility was confirmed, an in-person appointment was scheduled. During this appointment, details of the study were explained, participants' questions were answered, and informed consent was obtained.

### Measures

All study procedures and materials were approved by the university's Institutional Review Board. After providing informed consent, participants completed a 14-item paper survey, adapted from one designed by the West Virginia Office of Maternal, Child, and Family Health that assessed knowledge and awareness of HPV and Pap tests. Following completion of the survey, an in-depth, semistructured interview was conducted during the same session.

First author, Harb, a cisgender woman who conceptualized the study and conducted all interviews, asked a series of 10 open-ended questions about experiences with the health care system and the motivators and barriers for accessing health care services. The quantitative survey was not used as a discussion prompt during the qualitative interviews; however, we expected some discussion to focus on Pap testing as a key health care experience.

The overarching goal of interviews was to develop “thick” data on transgender/genderqueer AFAB individuals' health care experiences. Following Guba and Lincoln,^22^ thick data refer to obtaining sufficiently detailed information about a phenomenon so that analytic, rather than just descriptive, inferences can be made. As the study progressed, Pap testing emerged as a key experience that provided insight into the health care system and transgender/genderqueer AFAB individuals' use of services.

Participants were free to decline to answer any survey or interview question or could introduce new discussion topics during the interview as they wished. The interviewer was also free to deviate from the questions to ensure flow of conversation and to ask additional questions (i.e., probe) to construct a clear narrative of participants' sexual health care experiences.

Interviews were audio recorded and transcribed verbatim by the first author and a paid transcriptionist into Word Documents for analysis. To protect confidentiality, each participant was assigned a pseudonym and potentially identifying details were redacted from transcripts. There was no follow-up with the participants. Each participant was offered a $10 gift card to a department store as remuneration and a handout that included information about local supportive social and health care resources for transgender/genderqueer individuals.

### Analysis

Quantitative survey responses were entered into Excel documents, from which descriptive summaries (e.g., means of continuous variables, distributions of categorical variables, scores of multi-item scales) were calculated. Because of the small number of transcripts and a desire to stay close to the data, transcripts were coded manually as Word documents and codes were managed in Excel spreadsheets. Author Gilbert, who served as faculty supervisor of the study as a student research project, guided the process by developing a training protocol and overseeing the analysis. Authors Harb, Pass, and Zwick conducted a thematic analysis using an iterative process, meaning the qualitative data were systematically and repeatedly reviewed and coded.^23,24^

Led by author Harb, the analysts were trained to execute the same task of organizing the data in the same manner. The goal was to increase agreement among coders, and training consisted of preliminary coding exercises using draft codes followed by discussions until consensus was reached on code definitions and the coding process. Subsequently, each analyst coded interviews independently.

First, initial concept-driven (i.e., deductive) codes were created based on a preliminary literature review before interviews began.^1–14,16,17,19,20,25–27^ Second, data-driven (i.e., inductive) codes were derived after reading and coding transcripts. Both concept and data-driven codes were grouped under broadly salient topics, such as personal history (e.g., process of realizing gender identity), general health care utilization and experiences with specific services (e.g., Pap tests, STI screens), experiences with health care, attitudes toward health care, and general experiences in the community.

Codes were combined into thematic groups and meanings elaborated in several iterations. This was done initially by author Harb, who matched patterns of deductive and inductive codes to identify areas in which concept and data-driven themes overlapped and then drafted preliminary thematic definitions. All analysts contributed to revising or deleting codes and thematic groupings as they became more familiar with the data and the emerging narrative being constructed. Any coding discrepancies were discussed and resolved through consensus among all analysts.

Seeking to maximize the study's credibility, which corresponds to the positivist concept of validity,^28^ the research team followed practices described by Guba^29^ to create an audit trail of notes during analysis. These notes allowed the team to review decision points and reassess interpretations (e.g., the evolution of code definitions and thematic categories). Credibility was further ensured through triangulation of findings through internal and external processes. In the first case, the researcher team compared quantitative and qualitative responses on the same topic. In the second case, they compared emergent findings to published research reports on the same topic.

Dependability, the qualitative analog of the positivist concept of reliability,^28^ was achieved primarily through activities to maximize agreement among analysts as described above. In addition, co-author Gilbert undertook an inquiry audit throughout the duration of the study, checking the appropriateness of methods, and reviewing the audit trail (e.g., notes) for consistency of processes.^30^ A final credibility check consisted of each team member reviewing and revising the article to ensure its accuracy.

## Results

Seventeen individuals participated in the study, of whom nearly all (*n*=16) were recruited through the campus-wide e-mail. One participant was recruited through the medical center newsletter. None was recruited through snowball referrals.

Sample demographic characteristics are shown in Table 1. Participants were mostly young adults, ranging in age from 19 to 43 years, with a median age of 23 years. Five of 17 participants met the study's age eligibility criterion but had not yet reached the age of recommended Pap test utilization. Participants were nearly exclusively White, and largely (65%) students, likely reflecting the distribution of age and student status of the population reached by the campus-wide e-mail. All participants had medical insurance, with the most frequent types being coverage through their parents' plan (41%) or through a university plan (29%).

**Table 1.** Participant Characteristics (*n*=17)

	Mean±SD (min–max) or *n* (%), as appropriate
Age, years	26±8.1 (19–43)
Gender identity	
Transgender man	11 (65%)
Gender nonbinary	4 (24%)
Trans masculine	1 (6%)
Genderqueer	1 (6%)
Race/ethnicity	
White	16 (94%)
Latinx	1 (6%)
Employment status	
Student	11 (65)
Employed full- or part-time	5 (29)
Disabled	1 (6)
Insurance type	
On parent's insurance plan	7 (41)
Through university plan	5 (29)
Private insurance	3 (18)
Medicaid/Medicare	2 (12)

SD, standard deviation.

A majority of participants self-identified as transgender men (*n*=11), whereas minorities self-identified as gender nonbinary (*n*=4), trans masculine (*n*=1), or genderqueer (*n*=1). Six participants, all age 26 years or younger, mentioned that the internet helped them discover their gender identity, and four of those six specifically reported using amateur videos online. As Aiden (trans man, 20) recounted:

I knew something was up with my gender for a long time, right? but I had never heard of transgender. I just happened to come across somebody in a YouTube video talking about that, that he was a transguy, and I was like boom. I was just like, well, that explains it. So, that was kind of a Eureka moment.

Regarding knowledge of HPV, the average number of correct answers to the 14-item knowledge assessment was 10 (range 9–12). However, self-perceptions differed from quantitative results that suggested good knowledge. Despite generally high scores (Table 2), 41% of participants rated themselves as “unaware” and 59% rated themselves as “aware but not well informed” about HPV.

**Table 2.** Human Papillomavirus and Papanicolaou Test Knowledge Assessment (*n*=17)

Item	*n (%)*
HPV can be transmitted through the air	
Yes	0 (—)
No^a^	17 (100)
HPV is a sexually transmitted disease	
Yes^a^	17 (100)
No	0 (—)
HPV is a cause of cancer	
Yes^a^	17(100)
No	0 (—)
Condoms prevent all types of HPV	
True	0 (—)
False^a^	17 (100)
People still need to have routine Pap tests done if they have gotten the HPV vaccine	
Yes^a^	17 (100)
No	0 (—)
If you contract HPV, you will get genital warts	
True	4 (24)
False^a^	13(76)
At what age should people start getting Pap tests? (years)	
16	3 (18)
18s	6 (35)
21^a^	7 (41)
25	1 (6)
If you have contracted HPV, you will always present with symptoms	
True	0 (—)
False^a^	17 (100)
The Gardasil vaccine is only for people with female reproductive organs	
True	5 (36)
False^a^	9 (64)
You cannot get the vaccine if you are already sexually active	
True	0 (—)
False^a^	17 (100)
How many strains of HPV are there?	
4	4 (25)
25	4 (25)
40	3 (19)
100+^a^	5 (31)
How long does the Pap test itself take?	
A couple of minutes^a^	16 (94)
30 minutes	1 (6)
An hour	0 (—)
How often does a healthy person need to get a Pap test?	
Once a year	6 (35)
Once every 2 years	3 (18)
Once every 3 years^a^	8 (47)

^a^ Denotes correct response.

HPV, human papillomavirus; Pap, Papanicolaou.

The survey showed that 100% of the participants were aware that HPV is an STI that can cause cervical cancer and can oftentimes be asymptomatic. In addition, all 17 participants knew that routine Pap tests are still necessary after receiving HPV vaccination and that eligibility for the vaccine is possible even after a person is sexually active. Finally, every participant recognized that condoms could not prevent people from getting all types of the virus. Only 41% of respondents knew that the age for receiving routine Pap tests is 21, and eight participants (47%) correctly identified recommended Pap test frequency for a healthy person. Just over half (53%) knew that the HPV vaccine was for everyone, regardless of natal reproductive organs, and 5 participants (29%) knew that there are over 100 strains of HPV virus.

The qualitative interviews revealed that a large majority (88%) had previously received STI screenings, half (53%) of whom described it as a neutral experience. A slightly smaller proportion (40%) described it as a positive experience, and only one participant (7%) reported their STI screening experience as negative. Compared with STI screenings, a smaller majority of participants (59%) had previously received a Pap test, but a larger proportion of them (70%) described the Pap test as a positive experience. Two participants (20%) described it as a neutral experience, and one participant (10%) described the Pap test experience as negative.

Among those who had not received a Pap test, most (71%) were below the age of 21, which is the recommended age for routine screening. Nevertheless, four of these young participants (57%) stated that they would be willing to get a Pap test if a health care professional recommended it and gave them more information. In contrast, both participants who were 21 years or older but had not received a Pap test stated that although they understood the risks of HPV and had been urged by physicians to undergo a Pap test, they did not wish to do so.

There appeared to be no relationship between participants' age and distress related to seeking sexual health care. Altogether, eight (47%) participants expressed anxiety about Pap tests, but only two participants related it specifically to the procedure. In addition, there appeared to be no relationship between gender identity and distress. Those who reported distress self-identified as transgender, trans masculine, and nonbinary and used a range of pronouns. The anxiety associated with the Pap tests was almost always related to the fact that the participants did not identify as female.

The analysis of qualitative interview data identified one facilitating factor (health care provider's role and relationship) and three inhibiting factors (availability of transgender-competent care, distress about seeking sexual health care, and characteristics of the health care setting) regarding use of sexual health care services in general and receiving a Pap test in particular. Each key finding is detailed below.

### Health care provider's role and relationship

Health care providers appeared to be central in the decision-making process of whether or not to undergo Pap testing. Perhaps most obviously, patients expressed willingness to be tested if the health care provider recommended it, although they still expressed some reluctance. As Mason (trans man, age 20) explained:

I don't know if I would be proactive about it…but I just know that it's something to look out for. Yeah, I'm not opposed to the idea. If my doctor told me to go [for a Pap test], I would do it.

This sentiment was echoed by nine participants who had not received a Pap test, the majority of whom said they would agree to Pap testing with further education from their provider and a recommendation to get tested. Thus, health care providers may have considerable influence on transgender/genderqueer AFAB patients' utilization of services. Less obvious but equally important, health care providers were seen as responsible for setting a climate in which transgender/genderqueer AFAB patients could feel comfortable addressing their sexual health care.

Indeed, a number of participants who had received a Pap test said that they were more likely to do so because they had a prior positive relationship with the health care provider. However, participants clarified that it would take more than a willingness to see transgender/genderqueer AFAB patients; they wanted to have a health care provider who was comfortable with transgender patients and could make them comfortable in return. As Aiden (trans man, age 20) explained:

Most trans people can pick up if a doctor feels really uncomfortable because somebody is trans. So like…becoming comfortable with people being trans and seeing trans people is really important to making the trans person feel comfortable.

### Availability of transgender competent care

Despite high willingness to comply with recommendations and generally positive experiences, participants expressed frequent concern about finding clinics and health care providers that understood transgender/genderqueer AFAB individuals' health needs and could offer competent care. Accordingly, some participants described deliberately avoiding spaces that felt noninclusive, such as gynecology clinics. A number of participants favored primary care clinics, and general sexual health care clinics, especially ones that advertised lesbian, gay, bisexual, transgender and queer (LGBTQ) competent care. In fact, all seven patients who reported positive experiences with Pap tests reported having the test done in transgender inclusive settings (e.g., LGBTQ clinics), or with health care providers who specialized in transgender health care.

Alexander (trans man, age 28), who underwent a Pap test, considered the setting:

[The Pap test was] fine! I'm sure that it would be a little bit more uncomfortable for me if I presented more as a man and then ended up going to a gynecologist. That might be a little more awkward, but I just go to my family doc, and he does a fine job.

Some participants expressed doubt in their providers' ability to adequately treat transgender/genderqueer AFAB patients, especially after having initiated hormone replacement therapy. Others worried that health care providers in general were uneducated about transgender/genderqueer AFAB people's identities and did not know how to interact with them. As Danny (nonbinary, age 24) explained:

Every time I go in, there's always this, is it going to be awkward? Am I going to have to explain stuff again this time?

Other participants stated explicitly that uncertainty about competent care was a barrier to seeking a Pap test, as Ethan (trans man, age 23) stated:

I'm already reluctant to go to the doctor because I don't know the level of trans competence…For the last two years, every time I go to the doctor, they're like, “you need to get a Pap test.” I'm like, hmm, give me a trans competent doctor then.

In response, a frequently repeated recommendation was that clinics and health care providers explicitly advertise their competency with this population, for example on their clinic web profiles, which would be a step toward creating a welcoming clinical environment. As Aiden (trans man, age 20) explained:

I think that doctors who are comfortable with and affirming of trans people…it's a really good idea to put that on their profile page of their website or something like that. Because people are always looking for affirming physicians to go to.

Many participants stated that they had deliberately sought sexual health care at venues that promoted their services for transgender individuals or had received recommendations from transgender/genderqueer AFAB peers for trans masculine-friendly providers. Additionally, some participants suggested advertising specifically that Pap tests are not just for cisgender women. As Isaac (trans man, age 22) recalled, “in [city], they had posters that were like, ‘men get Pap smears too,’ or something like that. They were actually really cool.”

Of note, the study site was a university town that is home to an LGBTQ clinic affiliated with the university medical center; however, only one participant reported going there for a Pap test. Discussions revealed that the local LGBTQ clinic struggled to meet demand. Logistical barriers, such as limited hours and long waiting lists for appointments, prompted some participants to seek health care elsewhere. Nolan (trans man, 42) described his experience trying to make an appointment, saying “it was gonna be, like, six months, and at that point I was like, I'm not going to wait. So I actually went to Chicago.”

### Distress about seeking sexual health care

The juxtaposition of a masculine gender identity during a gynecological procedure was a powerful source of discomfort for some participants, as some participants anticipated being mistreated, treated differently because of their transgender identity, or having their gender invalidated. While participants noted that health care in general is highly binary (i.e., only male vs. female) and does not often allow adequate space for transgender identities in various ways, such as on health information forms, these concerns were often heightened in the context of seeking sexual health care. This results in considerable reluctance to seek sexual health care and avoidance of the Pap test altogether, despite knowledge of individual risk and consequences of HPV. As Grayson (trans masculine, age 22) states:

I know why you have to do it. HPV is a nasty virus, and a lot of people get it. It can cause cancer, and I know that…I know about the risks of this…[but] people's perceptions matter. I don't wanna walk into a situation in which I'm not going to be perceived as who I am.

This juxtaposition of a masculine gender identity and gynecological care caused a particularly sensitive situation for some participants and was noted by both those who had received the test and those who had not. Isaac (trans man, age 22) described the experience:

The process of getting a Pap smear itself is a little bit distressing…you have to put your feet in the stirrup things, and it feels like, ‘oh here I am in a gynecological examination.’…It's [an] incredibly vulnerable position that you're in. As nice as everybody is, it's just not a comfortable experience…especially if it's just, like, I'm coming and presenting as male, and oh yeah! Here's my vagina.

Indeed, the degree to which Pap tests can be distressing for transgender/genderqueer AFAB individuals was further exemplified by Noah's (nonbinary, 36) experience.

One time, when I went to get my Pap test, we were just talking about it, and I just broke down crying in the office. There's this profound sense of powerlessness. And I don't even know what I'm crying about, but it freaks me out.

### Characteristics of the health care setting

Beyond individual and interpersonal barriers, the health care setting was an important contextual factor. Several participants noted that clinics offering Pap tests were often female-oriented spaces that did not feel welcoming to transgender/genderqueer AFAB patients. Specifically, spaces were designed to appeal to cisgender women, implying that female natal reproductive organs were essential indicators of being a woman and negating the identity and experiences of transgender/genderqueer AFAB individuals. Participants noted that décor was the most visible expression of this orientation toward natal females. Lucas (trans man, age 26) described the waiting room of the clinic where he had undergone a Clinical breast exam and its effect on his intention to seek care.

Everything's pink. Very pink! And I had already started to look more masculine at that point, so I was uncomfortable. And I was surrounded by women. And when I went there, I immediately felt uncomfortable…I just didn't feel like I was welcome there. I wouldn't have gone back, even if it had been something bad. I would have searched for another clinic because that was really rough.

Noah (nonbinary, 36) also described feeling uncomfortable going to appointments because of the nature of the physical space, stating:

Part of it's probably that I don't want to be in a place where I just have to be [feminine]. And you know, the rooms are mauve and pink (laughing). I guess they can change that to green rooms or something. Mmm purple! I like purple. But…none of this mauve, pink, teal crap is doin' anything for me. It feels kind of alien I guess. And then it's all tied up with…pediatrics, and for obvious reasons, because it's reproductive health.

## Discussion

To inform future risk-reduction interventions and clinical practice, we undertook a study of the factors related to use of sexual health care services in general, and receipt of Pap tests in particular, by transgender/genderqueer AFAB individuals in a Midwestern state using a quantitative survey and qualitative interviews. As there is little existing research on transgender/genderqueer AFAB sexual health,^9,20^ particularly in rural areas, this study extends the geographic reach of the current evidence base.

Our findings about knowledge of and attitudes toward cervical cancer screenings are largely congruent with those identified in two previous qualitative studies.^14,31^ As those studies were conducted in a large coastal metropolitan area, our results obtained in a predominantly rural Midwest state suggest consistent salient themes across varying contexts. There may be a level of homogeneity among transgender/genderqueer AFAB individuals' experiences in health care settings.

However, two details are worth noting. A previous study by Agénor et al. included health care providers in their sample, yielding additional perspectives on the topic, whereas we restricted our sample to only transgender/genderqueer AFAB individuals. We look forward to future research that also explores Midwest and rural clinicians' knowledge of and attitudes toward transgender/genderqueer AFAB sexual health care.

In addition, both previous studies were conducted in a city with a well-recognized lesbian, gay, bisexual and transgender (LGBT) health care center. Although the present study took place in a town that is home to an LGBT clinic affiliated with a university medical center, few study participants had obtained sexual health care there and many cited logistical barriers, such as limited hours and long wait lists for appointments. Thus, the similarity of results is striking as our participants had obtained their health care from a wider variety of settings than peers in previous studies. We look forward to future studies that are able to explore this further, particularly focusing on experiences of transgender/genderqueer AFAB individuals in other Midwestern areas without established LGBT health care services, especially in rural and traditionally conservative settings.

Regarding knowledge, we noted a paradox—although the vast majority of participants scored highly on the survey, indicating that they had high levels of knowledge and awareness about HPV, not a single participant rated themselves as “aware and well informed” about the topic. This could have been due to a self-serving (i.e., face saving) bias; participants might have hesitated to rate themselves as well informed in case they answered some of the knowledge items incorrectly.

Nevertheless, the study showed that knowledge alone is not a sufficient motivator to seek health care services, a finding with broad applicability. Moreover, the items that had the fewest correct answers were generally about guidelines for Pap tests. This aligns with previous reports that neither patients nor providers are aware of recommendations for cervical cancer screenings for this population.^26^ Thus, neither health care providers nor their transgender/genderqueer AFAB patients may be able to properly assess the risk for HPV and other STIs.

Among themes that emerged from our interviews, the health care provider's role and relationship with patients was a facilitating factor for Pap test utilization. Other themes, including lack of transgender competent care, distress about seeking sexual health care, and unwelcoming characteristics of the health care setting, were inhibiting factors. Our finding of distress about seeking sexual health care is in alignment with both Dutton et al. earlier, and Peitzmeier et al. recent, qualitative studies,^27,31^ which suggest that for transgender/genderqueer AFAB individuals accessing and undergoing a Pap test is fraught by the juxtaposition between self-perceptions of gender identity and anatomy, which can heavily influence willingness to receive a Pap test.

Our findings indicate that emotional distress can be exacerbated by the actions, perceptions, and attitudes of health care providers, as well as the physical environment of the health care setting. Accordingly, transgender/genderqueer AFAB individuals may anticipate experiencing discrimination or gender-related discomfort while at the clinic, thus exacerbating resistance to cancer screening. However, we also note an opportunity for relief. Emotional distress could also be mitigated by skilled health care providers and inclusive clinic characteristics. Indeed, we see an opportunity for providers and clinics to play a greater role in helping transgender/genderqueer AFAB patients feel comfortable with Pap tests. We highlight the work of Potter et al.,^26^ which provides recommendations for inclusive sexual health care of trans masculine and noncisgender female identities, and look forward to continued professional education efforts to promote trans-competent health care.

Paralleling distrust of physicians, we noted a desire for further education among study participants. Specifically, of those who did not receive a Pap test, most said they would be willing to undergo testing with a health care provider's recommendation and additional information. To this end, we see great potential in novel attempts to reach transgender/genderqueer AFAB individuals directly, which could support community empowerment and complement ongoing professional education efforts for health care providers.

For example, future interventions could leverage social media technology to provide education and peer role modeling about sexual health care. We noted the use of online videos for gender identity exploration and envision popular transgender/genderqueer hosts of video channels serving as trusted opinion leaders and lay health advisors. Furthermore, social media can act as a virtual community, which may be particularly important in Midwest and rural states where there are few visible transgender/genderqueer peers and little sense of community. Emerging research has documented the central role of transgender peer videos^32^; however, to our knowledge there have been no evaluation studies of peer videos to disseminate health care information. This is an area ripe for intervention development.

Although the present study provides insights from an understudied geographical area, there are potential limitations to consider. First, the sample was nearly exclusively White, had a high level of education, and was comprised largely of students. Although it reflects the context of the Midwest university town where the study was conducted, findings may not reflect the experiences of transgender/genderqueer AFAB of color, older individuals, or those with less education.

Second, the study setting may have influenced results. Given the progressive social and political climate of the university town, motivators, barriers, and actual use of health care services may be different in other rural and micropolitan areas of the state.

Third, the qualitative methods employed in this study produced specific results. In other words, this study explored sexual health care knowledge, attitudes, and experiences in certain place, time, and sample. Findings may not apply to other transgender/genderqueer AFAB individuals; however, the details provided in this article will allow readers to assess transferability—the qualitative analog of generalizability^28^—to other populations for themselves.

In sum, this study identified key factors that facilitate or impede sexual health care for transgender/genderqueer AFAB individuals, confirmed that many factors identified in other geographical areas were also present in a Midwestern state, and provided local data to inform future health promotion interventions. Given the similarities between our findings and those reported by other researchers, there appears to be considerable homogeneity among transgender/genderqueer AFAB experiences in health care settings.

Although many barriers stand in the way of accessing care, one of the major causes of anxiety within this patient population is having a physician incompetent in transgender health issues. This perceived incompetence acts as a large barrier to pursuing preventive care. It is imperative that health care institutions take action by ensuring that clinicians are properly trained to accommodate needs of this vulnerable, and oftentimes neglected, population. Further research is warranted into the power dynamic experienced by transgender/genderqueer AFAB individuals during medical encounters, specifically during the Pap test. Finally, transforming the characteristics of the clinic setting to create a more gender-inclusive environment should be strongly considered.

## Abbreviations Used

ACOG: American College of Obstetricians and Gynecologists
AFAB: assigned female sex at birth
HPV: human papillomavirus
Pap: Papanicolaou
STI: sexually transmitted infections

## Notes

^*^ For further information on terminology, please see the online guide by the UCSF Center of Excellence for Transgender Health (http://transhealth.ucsf.edu/trans?page=guidelines-terminology), the Women's Health Research Network white paper *Better Science with Sex and Gender* (http://bccewh.bc.ca/wp-content/uploads/2012/05/2007_BetterSciencewithSexand GenderPrimerforHealthResearch.pdf), or the recently published book *Trans Bodies, Trans Selves* (ISBN: 9780199325351).
